# MRI-Based Intelligence Quotient (IQ) Estimation with Sparse Learning

**DOI:** 10.1371/journal.pone.0117295

**Published:** 2015-03-30

**Authors:** Liye Wang, Chong-Yaw Wee, Heung-Il Suk, Xiaoying Tang, Dinggang Shen

**Affiliations:** 1 School of Life Science, Beijing Institute of Technology, Beijing, 100081, China; 2 IDEA Lab, Department of Radiology and Biomedical Research Imaging Center (BRIC), University of North Carolina at Chapel Hill, Chapel Hill, NC 27599, United States of America; 3 Department of Brain and Cognitive Engineering, Korea University, Seoul, Republic of Korea; Banner Alzheimer's Institute, UNITED STATES

## Abstract

In this paper, we propose a novel framework for IQ estimation using Magnetic Resonance Imaging (MRI) data. In particular, we devise a new feature selection method based on an extended dirty model for jointly considering both element-wise sparsity and group-wise sparsity. Meanwhile, due to the absence of large dataset with consistent scanning protocols for the IQ estimation, we integrate multiple datasets scanned from different sites with different scanning parameters and protocols. In this way, there is large variability in these different datasets. To address this issue, we design a two-step procedure for 1) *first* identifying the possible scanning site for each testing subject and 2) *then* estimating the testing subject’s IQ by using a specific estimator designed for that scanning site. We perform two experiments to test the performance of our method by using the MRI data collected from 164 typically developing children between 6 and 15 years old. In the first experiment, we use a *multi-kernel* Support Vector Regression (SVR) for estimating IQ values, and obtain an average correlation coefficient of 0.718 and also an average root mean square error of 8.695 between the true IQs and the estimated ones. In the second experiment, we use a *single-kernel* SVR for IQ estimation, and achieve an average correlation coefficient of 0.684 and an average root mean square error of 9.166. All these results show the effectiveness of using imaging data for IQ prediction, which is rarely done in the field according to our knowledge.

## Introduction

Intelligent Quotient (IQ) is a score, which is generally derived from a variety of tests, to assess human intelligence. Although the test-takers show varying scores when taking the same test at different occasions or taking different tests at the same age, clinical psychologists in general regard IQ score as a statistically valid metric for clinical purposes [1,2]. However, the current standard IQ tests are not applicable to infants or young children because of their questionnaire-based test series. Should we develop a more systematic technique to estimate current IQ or to predict future IQ, it would hold great promises for identifying infants or young children who may undergo unusual intellectual development, thus providing a chance to conduct early interventions such as specialized and tailored educations for them.

Uncovering human intelligence has always been of major interest in cognitive neuroscience. With the advent of brain imaging, there have been efforts to investigate the relation between brain anatomy and intelligence [3,4], and substantial understanding has been achieved in the field. For example, Supekar *et al*. showed that the size and circuitry of certain parts of children’s brains could be a potential predictor for how well they would respond to intensive math tutoring [5]. Chen *et al*. [6] demonstrated that the volumetric analysis of gray matter (GM) from structural Magnetic Resonance Imaging (MRI) could be used to predict a subsequent decline in IQ in children with sickle cell disease. McDaniel *et al*. [3] found that the volume of the brain is positively correlated with IQ according to MRI-based experiments. Frangou *et al*. [7] reported positive correlations between IQ score and GM density of the orbitofrontal cortex, cingulate gyrus, cerebellum, and thalamus, but negative correlation between IQ score and the caudate nucleus. On the other hand, Navas-Sanchez *et al*. [8] investigated the relationship between IQ score and microstructure of white matter (WM) tracts using diffusion tensor imaging (DTI), and found that IQ score is positively correlated with fractional anisotropy (FA). Kim *et al*. [9] found that lower performance in verbal IQ score is correlated with the decrease of FA values. In another DTI-based study, Welcome *et al*. [10] discovered that the volume of WM fiber tracts is correlated with nonverbal IQ score. Inspired by these strong correlations between brain anatomy and IQ score, we propose, in this study, a novel framework to estimate IQ by using GM and WM features extracted from structural MRI.

In the proposed framework, a machine learning technique is particularly designed to better estimate IQ score of a testing subject. Here, we treat the IQ estimation as a regression problem by taking the GM and WM features derived from MRI images as predictors and the corresponding IQ scores as target responses. However, in the context of neuroimaging data analysis, one of the most crucial and challenging issues is to build a generalized model for the cases with high feature dimensionality and small sample size [11–13]. Dimensionality reduction or feature selection has been considered as a promising approach to circumvent this limitation. While the former finds a new low-dimensional space to which the features in an ambient space are projected, the latter selects task-related features in the original feature space. Therefore, it is in general more natural and intuitive for a feature selection approach to interpret and understand the results. Hence, we pursue the feature selection strategy in this work.

The existing feature selection methods can be broadly categorized into three types: filter-based, wrapper-based, and embedded-based approaches [11,14]. The filter-based approach selects subsets of features as a pre-processing step, but often ignores interaction among selected features. On the other hand, the wrapper-based approach uses a certain function to rank subsets of features according to their predictive power, but usually requires a huge computational cost. The embedded-based approach performs feature selection during optimization process, and is specific to the corresponding classification method. This approach usually proceeds more efficiently by directly optimizing a two-part objective function, with a goodness-of-fit term and another penalty term, for selection of a large number of variables. This also means that we can develop feature selection methods by simply adjusting the penalty term in the objective function. Thus, in this paper, we focus on the embedded-based feature selection approach.

Recently, multi-task learning based feature selection methods have attracted increasing attention in machine learning, computer vision and artificial intelligence [15–19]. A task is usually referred to feature selection for a modality or for a type of target responses. Multi-task learning utilizes the intrinsic relationship among different tasks during a learning process [20,21], and thus achieved better performances than the counterpart single-task learning method, i.e., learning each task separately. Specifically, recent emergence of sparse least square regression method penalized by a *L*_2,1_-norm regularizer, called group sparse learning, allows us to select variables that can be jointly used for multiple tasks [17,22]. Hereafter, we use the terms of “variable” and “feature” interchangeably. The main limitation of the group sparse learning arises from its strong assumption that different tasks should share the same features, which often contradicts with the real situations, without considering the task-specific characteristics [23]. To mitigate this limitation, Jalali *et al*. [24] proposed a dirty model by integrating a *L*_1_-norm regularizer so that different tasks could share the same features but still have chance to preserve their respective characteristics. Concretely, this model decomposes the weight coefficient matrix into two parts, i.e., group-wise feature sparsity and element-wise feature sparsity. Note that the *L*_1_-norm based regularization tends to randomly select only a single feature from a group of highly correlated features [25]. Since the dirty model uses a *L*_1_-norm based regularization, it has the same problem.

In this paper, we propose a novel feature selection method by extending the dirty model. Specifically, we devise a new regularization term with a squared Frobenius norm of the element-wise sparsity matrix to circumvent the problem of randomly selecting one feature from a group of highly correlated features.

In this study, we treat feature selections for WM and GM features, with a shared target such as IQ score, as two different tasks. Thus, multi-task feature selection can be used in our application of IQ estimation with selected WM and GM features [21]. According to Reiss’s report, age is correlated to brain tissue volumes [26]. Thus, we also study the effect of age on our estimators in a supplementary experiment.

The remainder of the paper is organized as follows. In the Materials and Preprocessing section, we provide information on the image data and the preprocessing pipeline. Then, the mathematical detail of the proposed feature selection method is described in the Method section. Finally, in the Experiment and Results section, we demonstrate the validity of the proposed method in estimating IQs with MRI image features by comparing with the state-of-the-art methods. Finally, we discuss our findings and conclude our work in the Discussions and Conclusion section, respectively.

## Materials and Preprocessing

### Subjects

We downloaded the data from Autism Brain Imaging Data Exchange (ABIDE) (available at http://fcon_1000.projects.nitrc.org/indi/abide/). Specifically, we used MRI samples of 164 (male/female: 130/34) typically developing children between 6 and 15 years old (11.1±2.1). MR images were scanned at 5 different sites: New York University Langone Medical Center (NYU: 59 samples), Kennedy Krieger Institute (KKI: 31 samples), Stanford University (Stanford: 20 samples), Oregon Health and Science University (OHSU: 15 samples), and University of California at Los Angeles (UCLA: 39 samples), using different scanning parameters and protocols. Concisely, two different datasets (each with 26 and 13 respectively) were scanned at UCLA. But, due to the limited number of samples, in this paper, we considered them as being from one site. Also, due to relatively small numbers of samples from Stanford and OHSU, we combined them and considered as a ‘SOHSU’ dataset of 35 samples. **Table 1** summarizes the demographic characteristics of subjects used in this paper.

**Table 1:** Demographic characteristics of the used subjects. For age and IQ scores, we show the mean and corresponding standard deviations (SD).

Data sets	Age (mean ± SD)	IQ scores (mean ± SD)	Male/female
NYU	11.3 ± 2.4	114.3 ± 13.6	42/17
KKI	10.2 ± 1.2	114.2 ± .9	24/7
SOHSU	10.0 ± 1.4	113.6 ± 13.4	31/4
UCLA	12.4 ± 1.4	107.4 ± 11.3	33/6

### Data Acquisition and Preprocessing

For the details of data protocols and scanning parameters, please refer to ‘http://fcon_1000.projects.nitrc.org/indi/abide/’. Since the data used in this paper is publicly available, it does not require any ethics statement. For MR images, we performed image preprocessing by following the common pipeline of skull stripping [27], cerebellum removal, tissue segmentation (into gray matter (GM), white matter (WM), cerebrospinal fluid (CSF)), and registration to a template. For the registration, we used HAMMER [27,28], which have been successfully applied to a variety of datasets. We used the anatomical automatic labeling (AAL) atlas with 90 predefined regions. We then computed GM and WM tissue volumes of each of the 90 regions and used them as features, i.e., 90 GM features and 90 WM features.

## Methods

**Fig 1:**
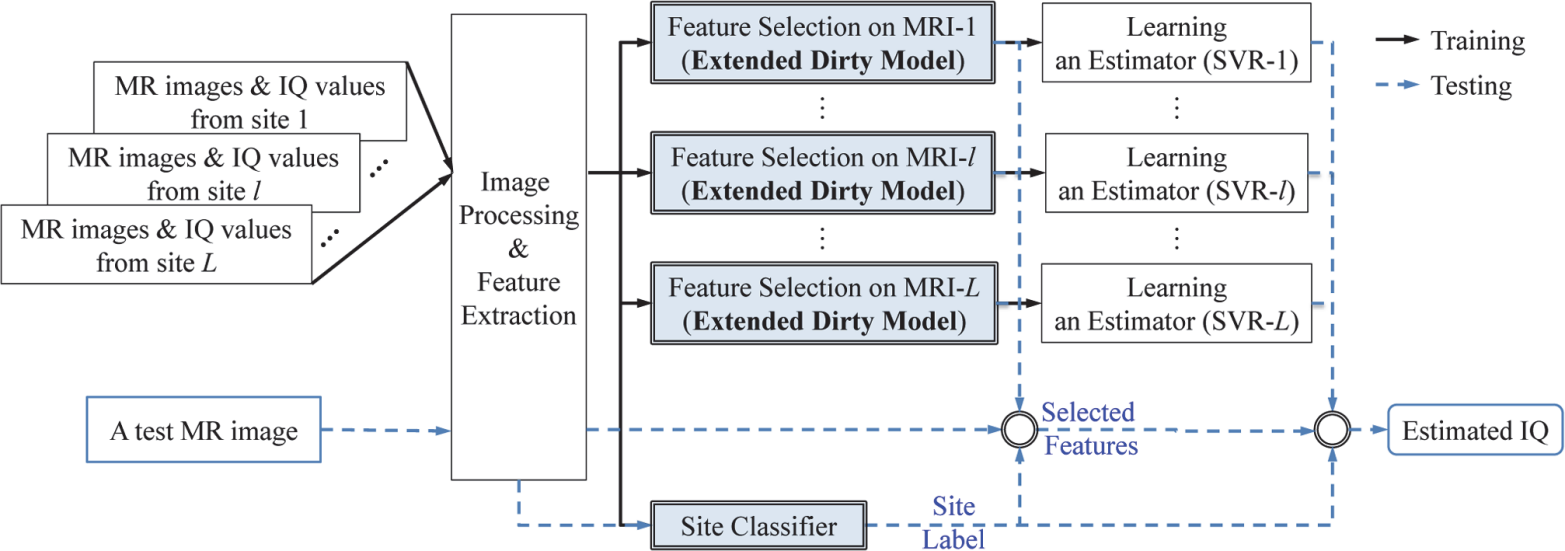
A schematic diagram of the proposed IQ estimation framework using structural MRI data.

In this section, we propose a novel framework for IQ estimation using structural MRI features. As explained in the section of Data Acquisition and Preprocessing, the MRI datasets used in this paper were obtained from multiple imaging centers with different scanning parameters and protocols. Hence, there exists an inevitable high inter-dataset variability. For this reason, we use a two-step procedure in our framework as shown in **Fig. 1**. Specifically, given the MR images scanned at multiple scanning sites and their respective IQs, we first extract two types of imaging features, i.e., GM volumes and WM volumes, by going through the image preprocessing procedure as described above. We then select informative features with the proposed extended dirty model (which will be described below) to build an IQ estimator using a Support Vector Regression (SVR) model [20]. Here, it should be noted that the feature selection and SVR model learning are performed independently for different datasets. That is, for our four datasets, we will have their respective selected feature sets and SVRs. Besides feature selection models and estimators, we also construct a classifier to identify the scanning site at which a MR image was scanned. In the testing phase, given a testing MR image, we first perform the same procedures of image preprocessing and feature extraction, and then feed the extracted features to the *site classifier* to identify the scanning site. It is worth noting that the testing samples are not restricted to the predefined sites. Actually, for any given sample even from an unknown site, the *site classifier* can assign it to a site whose data is most similar to the testing sample. Based on the identified site (labeled as *l* in Fig. 1), we can finally estimate the testing subject’s IQ score by using the corresponding selected feature set and SVR estimator (SVR-*l*). It should be noted that, due to the lack of available longitudinal data, in this work, we only focus on the *estimation* of the current IQ score, not the *predication* of the future IQ score, but the proposed framework can be extended to predict a subject’s future IQ score.

In the following, we will first describe the proposed feature selection method along with the training of an IQ score estimator, followed by a classifier to identify MRI data scanning site. Throughout the paper, we denote matrices, vectors, and scalars as boldface uppercase, boldface lowercase, and normal italic letters, respectively, and use a superscript *T* for a vector/matrix transpose.

### Feature Selection via Extended Dirty Model

Due to the relatively small number of samples compared to the feature dimensionality, it is of importance to reduce the dimensionality for avoiding the over-fitting problem. Among various dimensionality reduction methods, in this paper, we focus on using the popular sparse least squared regression method, which has been successfully applied to diverse applications [20,29,30]. For clarity and simplicity, let us omit a notation of a scanning site; but we should note that, in this paper, the feature selection method described below is applied independently to the dataset of each scanning site.

Hereafter, let us denote *G* and *W* for GM and WM, respectively. Let $X^{\left(G\right)}={\left[x_{n}^{\left(G\right)}\right]}_{n=1}^{N}\in R^{D\times N},X^{\left(W\right)}={\left[x_{n}^{\left(W\right)}\right]}_{n=1}^{N}\in R^{D\times N}$ and $y={\left[y_{n}\right]}_{n=1}^{N}\in R^{N}$ denote, respectively, a set of *D*-dimensional feature vectors from GM, a set of *D* -dimensional feature vectors from WM, and the respective IQ scores of *N* subjects. In this paper, we assume that the target IQ scores **y** can be represented by a linear combination of the features, i.e., GM features **X** ^(G)^ and WM features **X** ^(W)^, as follows:

$$y=X^{{\left(G\right)}^{T}}w^{\left(G\right)}+e^{\left(G\right)}$$ (1)

$$y=X^{{\left(W\right)}^{T}}w^{\left(W\right)}+e^{\left(W\right)}$$ (2)

Where **w** ^(G)^ ∈ *R* ^*D*^ and **w** ^(W)^ ∈ *R* ^*D*^ denote weight coefficient vectors of the respective feature vectors, and **e** ^(G)^ ∈ *R* ^*N*^ and **e** ^(W)^ ∈ *R* ^*N*^ are the noise vectors drawn independently from a standard Gaussian distribution.

Since we parcellate a human brain into multiple regions and extract regional GM/WM tissue volume features, it is natural to assume the existence of a shared structure between two feature types, and thus group lasso [22] can be used:

$$\underset{W}{\min}L\left(W\right)=\frac{1}{2}\sum_{i\in \{G,W\}}{\left\|y-X^{{\left(i\right)}^{T}}w^{\left(i\right)}\right\|}_{2}^{2}+\lambda {\left\|W\right\|}_{1,2}$$ (3)

Where **W** = [**w** ^(G)^ **w** ^(W)^]∈ *R* ^*D×2*^, and *λ* is a regularization parameter. It is, however, too strong to leverage the parameter overlap across all the features by means of group lasso [24,31]. Meanwhile, we believe that it is reasonable to use a dirty model [24] that can efficiently formulate the regularization scheme of 1) penalizing parameter overlap when it exists and 2) not penalizing parameter overlap when it doesn’t exist by using two separate parameter sets as follows:

$$\begin{array}{c} \underset{P,Q}{\min}L\left(P,Q\right)=\frac{1}{2}\sum_{i\in \{G,W\}}{\left\|y-X^{{\left(i\right)}^{T}}w^{\left(i\right)}\right\|}_{2}^{2}+\lambda_{1}{\left\|P\right\|}_{1}+\lambda_{2}{\left\|Q\right\|}_{1,2}\\ s.t.\;W=P+Q \end{array}$$ (4)

where **P** ∈ *R* ^*D×2*^ and **Q** ∈ *R* ^*D×2*^ are two parameter matrices that encourage element-wise sparsity and group-wise sparsity, respectively.

**Fig 2:**
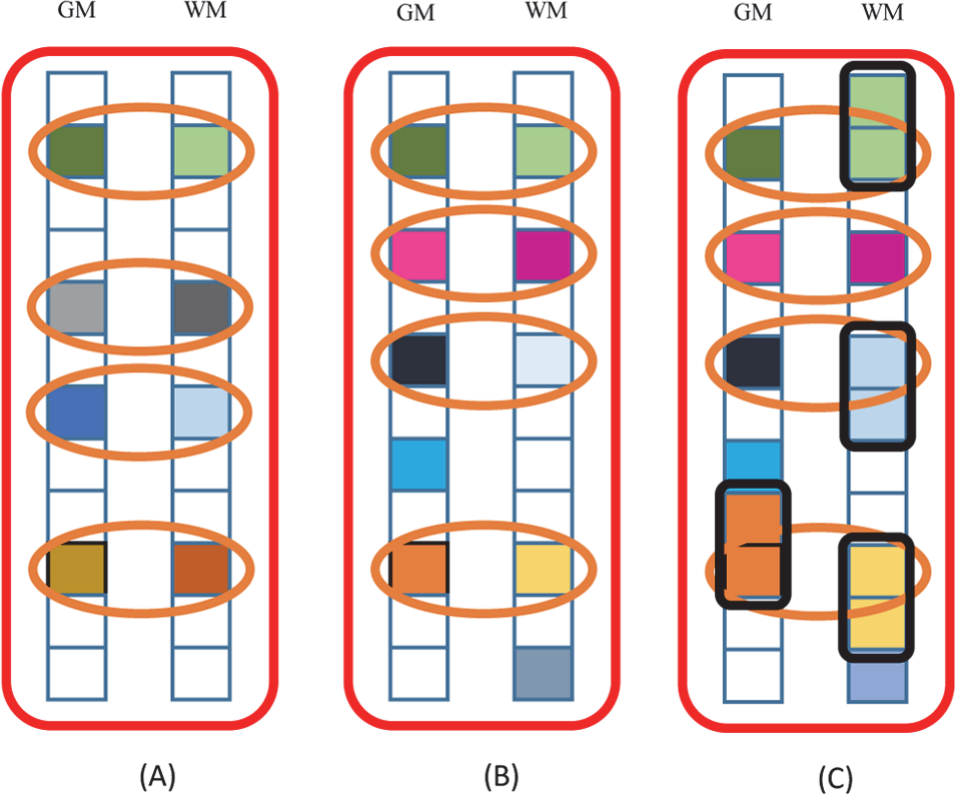
Comparison of weight coefficient matrices for three different feature selection methods. Each colored square corresponds to a non-zero element after feature selection. Circled squares (with the yellow ellipse outlines) correspond to the selected group-wise features, and circled squares (with black rectangle outlines) correspond to the selected pair-wise correlated features. (A) Group lasso. (B) Traditional dirty model. (C) The proposed extended dirty model.

However, it is known that the solution of **P** for the element-wise sparsity tends to randomly select one feature from a group of highly correlated features. To this end, we propose to extend the original dirty model by further regularizing the parameter matrix **P** with a squared Frobenius norm as follows:

$$\begin{array}{c} \underset{P,Q}{\min}L\left(P,Q\right)=\frac{1}{2}\sum_{i\in \{G,W\}}{\left\|y-X^{{\left(i\right)}^{T}}w^{\left(i\right)}\right\|}_{2}^{2}+\lambda_{1}{\left\|P\right\|}_{1}+\lambda_{2}{\left\|Q\right\|}_{1,2}+\lambda_{3}{\left\|P\right\|}_{F}^{2}\\ s.t.\;W=P+Q \end{array}$$ (5)

where ‖⋅‖_*F*_ denotes a Frobenius norm. In this paper, we call this new model as ‘extended dirty model’. By combining the relaxations of ‖**P**‖_1_ and ${\left\|P\right\|}_{F}^{2}$ in our objective function, we can jointly select the highly correlated features, but still encourage the group-wise feature selection, i.e., jointly selecting or unselecting regional GM/WM features, because of the *L*_2,1_-norm penalization on **Q**, i.e., ‖**Q**‖_1,2_. In this way, we can efficiently handle *not only* the shared inter-feature-type structure, *but also* the pairwise intra-feature-type correlations (as shown in **Fig. 2**).

After solving the optimization problem in Eq. (4) via an accelerated proximal gradient method [32–34], we select the informative GM and WM features based on the non-zero entries of the respective weight coefficient vectors **W** = [**w** ^(G)^ **w** ^(W)^].

#### Multi-Kernel Support Vector Regression

The selected features are then fed into a multi-kernel support vector regression (SVR) model [20], in which we fuse the complementary information of the two feature types, i.e., GM and WM volumes. After feature selection, given dimension-reduced *N* training samples ${\left\{{\hat{x}}_{n}^{\left(G\right)},{\hat{x}}_{n}^{\left(W\right)}\right\}}_{n=1}^{N}$ along with the corresponding target response${\left\{y_{n}\right\}}_{n=1}^{N}$, the multi-kernel SVR solves the following primal formulation that uses the *ε*-insensitive loss function:

$$\begin{array}{l} \underset{w^{\left(G\right)},w^{\left(W\right)},b,\xi ,\xi^{*}}{\min}\frac{1}{2}\sum_{i\in \left\{G,W\right\}}\beta^{i}w^{{\left(i\right)}^{2}}+C\sum_{n=1}^{N}\left(\xi_{n}+\xi_{n}^{*}\right)\\ s.t.\{\begin{array}{c} \sum_{i\in \left\{G,W\right\}}\beta^{i}\left({\left(w^{\left(i\right)}\right)}^{T}\phi^{\left(i\right)}\left({\hat{x}}_{n}^{\left(i\right)}\right)+b\right)-y_{n}\leq \varepsilon +\xi_{n};\\ y_{n}-\sum_{i\in \left\{G,W\right\}}\beta^{i}\left({\left(w^{\left(i\right)}\right)}^{T}\phi^{\left(i\right)}\left({\hat{x}}_{n}^{\left(i\right)}\right)+b\right)\leq \varepsilon +\xi_{n}^{*}\\ \xi_{n},\xi_{n}^{*}\geq 0,\;n=1,2,\ldots ,N \end{array} \end{array}$$ (6)

where **w** ^(G)^ and **w** ^(W)^ are the weight vectors, *ϕ* ^(G)^ and *ϕ* ^(W)^ denote the kernel-induced mapping functions of the two feature types (GM and WM), *β* ^*i*^ is a mixing coefficient with the constraint of *β* ^*i*^ ≥ 0 and ∑_*i*∊{G, W}_ *β* ^*i*^ = 1, *ξ*_*n*_ and $\xi_{n}^{*}$ are the two sets of slack variables, and *b* is a bias. We then derive the dual function form of the multi-kernel SVR as follows:

$$\begin{array}{c} \underset{\alpha ,\alpha^{*}}{\max}\;-\frac{1}{2}\sum_{n,m=1}^{N}\left(\alpha_{n}^{*}-\alpha_{n}\right)\left(\alpha_{m}^{*}-\alpha_{m}\right)\sum_{i\in \left\{G,W\right\}}^{}\beta^{i}k^{i}\left({\hat{x}}_{n}^{\left(i\right)},{\hat{x}}_{m}^{\left(i\right)}\right)\\ -\varepsilon \sum_{n=1}^{N}\left(\alpha_{n}^{*}+\alpha_{n}\right)+\sum_{n=1}^{N}\left(\alpha_{n}^{*}-\alpha_{n}\right)y_{n}\\ s.t.\;\sum_{n=1}^{N}\left(\alpha_{n}-\alpha_{n}^{*}\right)=0\;\text{and}\;0\leq \alpha_{n},\alpha_{n}^{*}\leq C,n=1,2,\ldots ,N \end{array}$$ (7)

where $k^{i}\left({\hat{x}}_{n}^{\left(i\right)},{\hat{x}}_{m}^{\left(i\right)}\right)={\left[\phi^{\left(i\right)}\left({\hat{x}}_{n}^{\left(i\right)}\right)\right]}^{T}\phi^{\left(i\right)}\left({\hat{x}}_{m}^{\left(i\right)}\right)$ is the kernel function of the two training subjects in the feature type *i*, and ${\left\{\alpha_{n},\alpha_{n}^{*}\right\}}_{n=1}^{N}$ are Lagrangian multipliers. We use a weighted linear combination of the kernel matrices as follows:

$$K\left[\left({\hat{x}}_{n}^{\left(G\right)},{\hat{x}}_{n}^{\left(W\right)}\right),\left({\hat{x}}^{\left(G\right)},{\hat{x}}^{\left(W\right)}\right)\right]=\sum_{i\in \left\{G,W\right\}}\beta^{i}k^{i}\left({\hat{x}}_{n}^{\left(i\right)},{\hat{x}}^{\left(i\right)}\right)$$ (8)

where $\left({\hat{x}}_{}^{\left(G\right)},{\hat{x}}_{}^{\left(W\right)}\right)$ is a new dimension-reduced testing subject. In this paper, we use a polynomial function for$\left(\phi^{\left(G\right)},\phi^{\left(W\right)}\right)$. After training a multi-kernel SVR, we can estimate a testing subject’s IQ as follows:

$$f\left({\hat{x}}^{\left(G\right)},{\hat{x}}^{\left(W\right)}\right)=\sum_{n=1}^{N}\left(\alpha_{n}^{*}-\alpha_{n}\right)K\left[\left({\hat{x}}_{n}^{\left(G\right)},{\hat{x}}_{n}^{\left(W\right)}\right),\left({\hat{x}}_{}^{\left(G\right)},{\hat{x}}_{}^{\left(W\right)}\right)\right]+b$$ (9)

### Construction of Site Classifier

Due to the inevitable inter-dataset variability caused by varying scanning parameters and protocols across different scanning sites, we propose to construct a site classifier for identifying the scanning site at which a testing MR image was scanned. Specifically, we use a sparse multinomial logistic regression (SMLR) model formulated as follows:

$${\hat{Z}}_{\text{MAP}}=\underset{Z}{\mathrm{arg max}}\left[\sum_{n=1}^{N}\left(\sum_{l=1}^{L}c_{n}^{\left(l\right)}z^{{\left(l\right)}^{T}}x_{n}-\log\sum_{l=1}^{L}\exp\left[z^{{\left(l\right)}^{T}}x_{n}\right]\right)+\log p(Z)\right]$$ (10)

where $x_{n}=\left[x_{n}^{\left(G\right)};x_{n}^{\left(W\right)}\right]$ is an augmented feature vector that concatenates the original two (GM and WM) feature vectors of the *n*-th training sample, **z** ^(*l*)^ is a weight vector for the scanning site *l*, *p*(**Z**) is a prior on the parameter matrix $Z={\left[z^{\left(l\right)}\right]}_{l=1}^{L}\in R^{D\times L}$, *L* is the total number of scanning sites, and $c_{n=}{\left[c_{n}^{\left(l\right)}\right]}_{l=1}^{L}\in R^{L}$ is a site label of the *n*-th sample, represented by a “1-of-*L*” encoding vector such that $c_{n}^{\left(l\right)}=1$ if **x**_*n*_ belongs to the scanning site *l* and $c_{n}^{\left(l\right)}=0$ otherwise. In this paper, *l* ∊ {NYU, KKI, UCLA, SOHSU} and *L* = 4. Regarding the prior *p*(**Z**), we use a Laplacian function (*p*(**Z**) = exp[−*γ*‖**Z**‖_1_], where *γ* is a sparsity control parameter) that is most widely used in the literature. The rationale of using SMLR as our classifier is that, unlike other classifiers, it automatically selects class-discriminative features and learns a separating hyper-plane. Please refer to [35] for a detailed explanation on SMLR.

## Experimental Results

To validate the effectiveness of our method in estimating a subject’s IQ score by using neuroimaging data, we perform extensive experiments and also compare our feature selection method with state-of-the-art methods, i.e., dirty model, group lasso, and elastic net [25]. Note that, the dirty model can select both group-wise and element-wise features, while group lasso only selects group-wise features. Elastic net is a single task learning method that can select element-wise features, and at the same time encourage the selection of pair-wise correlated features.

### Experimental Settings

We performed experiments with 10-fold cross-validations. Specifically, we randomly partitioned each dataset into 10 subsets with no replacement, and used 9 out of the 10 subsets for training and the remaining one for testing. To further avoid a possible bias during partitioning, we repeated the experiments 10 times. Note that, in each experiment, we built one site classifier to identify the scanning site where a test MR image was acquired, and also constructed four IQ score estimators, i.e., one multi-kernel SVR for each scanning site. Specifically, for training the site classifier, the training samples of all the datasets were used together, but for training the IQ score estimators for different scanning sites, only the training samples of the respective dataset were used. It is also worth noting that the process of training site classifier is independent from that of feature selection and training for regression models.

We used a degree-2 polynomial kernel function for multi-kernel SVR. For determining the model parameters, i.e., *λ*_1_, *λ*_2_, and *λ*_3_ in Eq. (4), kernel parameters *c*, *p* and weights *β* in multi-kernel SVR, and a sparsity control parameter *γ* in SMRL, we further divided the training samples for inner cross-validation and then obtained the optimal parameter set that produced the best performance in the inner loop. These parameter values are finally used for the left-out testing samples [13].

We considered three experimental scenarios as follows:

- Multi-kernel SVR based estimation: We compare the proposed method with three different feature selection methods, namely, 1) dirty model, 2) group lasso, and 3) elastic net. In our work, we regard finding the optimal weight vectors for GM and WM features (for estimating the target IQ score) as two tasks. The first two methods correspond to multi-task learning that jointly considers multiple tasks, while the last one corresponds to single-task learning. Therefore, for the elastic net, we select features of GM and WM independently. Then, multi-kernel SVR is used to combine the selected GM and WM features for IQ score estimation. In the experiment, for all competing methods, we use the same SMLR-based classifier for identifying the scanning site and also the same multi-kernel SVR-based estimator.
- Effect of age to the estimation with multi-kernel SVR: According to [26], age is correlated to the relative brain tissue volume and IQ. Thus we investigated the effect of age on our estimators by including it as an additional feature in a supplementary experiment. Specifically, we compute its kernel matrix, by assigning a small weight to it, i.e., 0.2, and then linearly combine it with the existing GM and WM kernel metrics, which are computed using the features selected by all competing methods. In the experiment, we use the same SMLR-based site classifier and also the same multi-kernel SVR-based estimator for all competing methods.
- Single-kernel SVR based estimation: We validate the efficacy of the multi-kernel approach by comparing with the single-kernel approach. The main difference between these methods lies in the fact that, while the single-kernel method assigns a uniform weight for different feature types, i.e., GM and WM, the multi-kernel method finds the optimal weight for each feature type based on the training samples. Specifically, for the dirty model, group lasso, and the proposed extended dirty model, we concatenate the selected features into a long vector and then fed them into a single-kernel SVR. For elastic net, we concatenate the GM features and WM features before feature selection, and then feed the selected features into a single-kernel SVR. Similar to the first experiment, we used the same SMLR-based classifier for identifying the scanning sites, and the same single-kernel SVR for all competing methods.

### Site Classification Results

**Fig 3:**
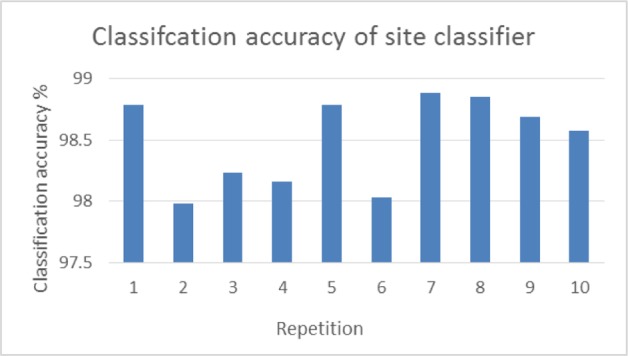
Classification accuracy of site classifier for each repetition, where a 10-fold cross-validation was performed.

Due to high variability of the inter-dataset caused by different scanning protocols and parameters, it is natural to build IQ score estimators that are optimized to the respective scanning site. In this regard, we first need to identify the scanning site of the given testing image in order to select the appropriate features and respective IQ score estimator. **Fig. 3** shows the performance of site classifiers, which are repeated 10 times with an averaged classification accuracy achieving 98.5%, in each of which a 10-fold cross-validation was performed.

### Multi-kernel SVR-based IQ Score Estimation Results

**Fig 4:**
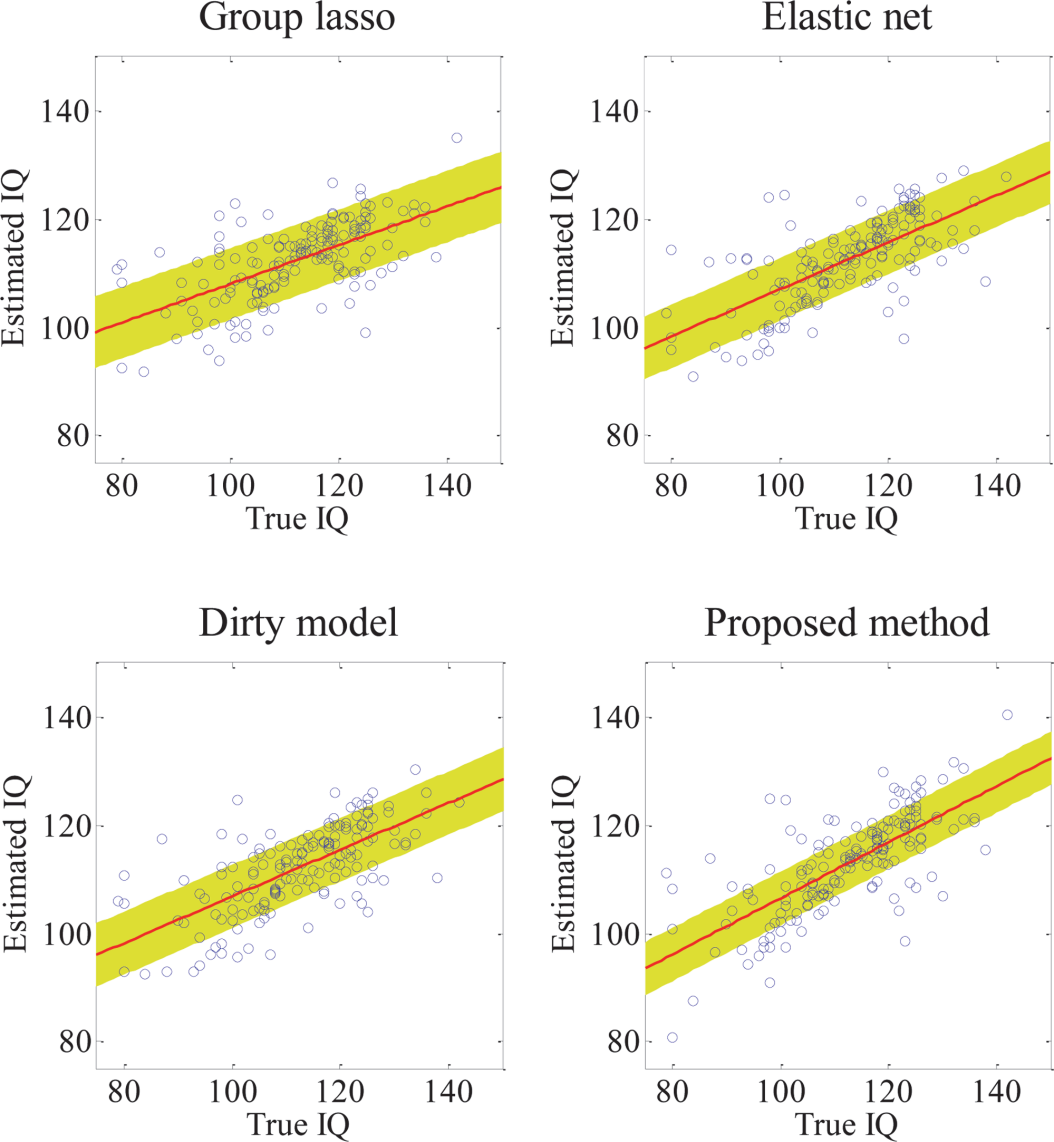
Scatter plots of the true IQ vs. the estimated IQ by multi-kernel SVR. Scatter plots of the true IQ vs. the estimated IQ by the four competing methods with multi-kernel SVR, along with the standard deviation of the distance for each point to the fitted line.

In Table 2, we presented the performances of all the competing methods using 1) the metrics of correlation coefficients (CC) and 2) the root mean square errors (RMSE) between the true IQ scores and the estimated ones. For methods of using both feature types, i.e., WM and GM, the best CC was 0.718 by the proposed method, while the other competing methods achieved 0.622 (group lasso), 0.682 (elastic net), and 0.68 (dirty model), respectively. In the meantime, the proposed method also produced the least RMSE of 8.695, outperforming other competing methods: 9.822 (group lasso), 9.145 (elastic net), and 9.182 (dirty model). The scatter plots of the true IQ scores vs. the estimated IQ scores by all competing methods are presented in **Fig. 4**.

**Table 2:** Performance (mean ± standard deviation) comparison among all competing methods in both experiments.

	Group lasso	Elastic net	Dirty model	Proposed method
CC	0.622 ± 0.005	0.682 ± 0.009	0.68 ± 0.012	**0.718** ± **0.006**
RMSE	9.822 ± 0.045	9.145 ± 0.092	9.182 ± 0.121	**8.695** ± **0.075**
	A-Group lasso	A-Elastic net	A-Dirty model	**A-Proposed method**
CC	0.621 ± 0.027	0.677 ± 0.012	0.685 ± 0.018	**0.726** ± **0.012**
RMSE	9.780 ± 0.263	9.200 ± 0.136	9.114 ± 0.194	**8.609** ± **0.146**

The prefix ’A’ denotes the use of age as a kernel matrix. (CC: Correlation Coefficient; RMSE: Root Mean Square Error)

### IQ Estimation Results of Multi-kernel based SVR with Age

**Table 2** also shows the results of all competing methods using age as additional feature, with a prefix ‘A’ in the front. The best correlation coefficient (CC) was 0.726 by *A-Proposed* method, while the other competing methods achieved 0.621 (*A-Group lasso*), 0.677 (*A-Elastic net*) and 0.685 (*A-Dirty model*). At the same time, the *A-Proposed* method produced the least RMSE of 8.609, which is superior to 9.78 (*A-Group lasso*), 9.2 (*A-Elastic net*) and 9.114 (*A-Dirty model*). In **Table 3**, we also added a prefix ‘t’ in the front of each method to denote the pair-wise t-test for CC and RMSE between the methods with age and the corresponding method without using age. The *p*-value of CC is 0.791 (t-Group lasso), 0.376 (t-Elastic net), 0.376 (t-Dirty model) and 0.136 (t-Proposed method). In the meantime, the *p*-value of RMSE is 0.643 (t-Group lasso), 0.359 (t-Elastic net), 0.302 (t-Dirty model) and 0.11 (t-Proposed method). These results actually show the use of age did not significantly improve the performance.

**Table 3:** The prefix ’t’ denotes the t-test for CC and RMSE between the methods with age and the corresponding method without using age.

	t-Group lasso	t-Elastic net	t-Dirty model	t-Proposed method
*p*-CC	0.791	0.376	0.376	0.136
*p*-RMSE	0.643	0.359	0.302	0.110

The prefix ’*p*’ denotes *p* value.

### Selected Brain Regions for IQ Score Estimation

**Fig 5:**
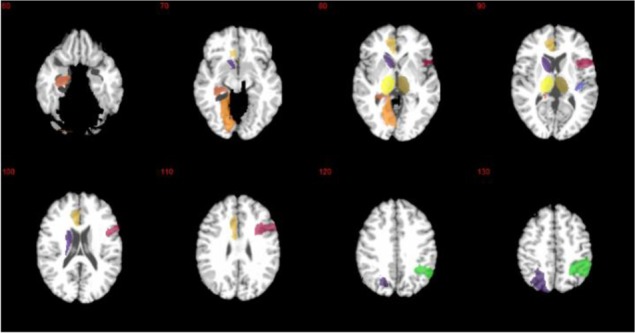
The 15 most frequently selected brain areas by the proposed method. Colors mainly show different regions.

**In Fig. 5**, we marked the 15 brain areas, of which features were most frequently selected by the proposed method to estimate IQ scores. Those brain areas include left/right transverse temporal gyri, left/right thalamus, left parahippocampal gyrus, left hippocampus, right opercular part of inferior frontal gyrus, left anterior cingulate gyrus, right amygdala, left lingual gyrus, left superior parietal lobule, right inferior parietal lobule, left angular gyrus, left paracentral lobule, and left caudate nucleus.

### Single-Kernel SVR-based IQ Score Estimation Results

**Fig 6:**
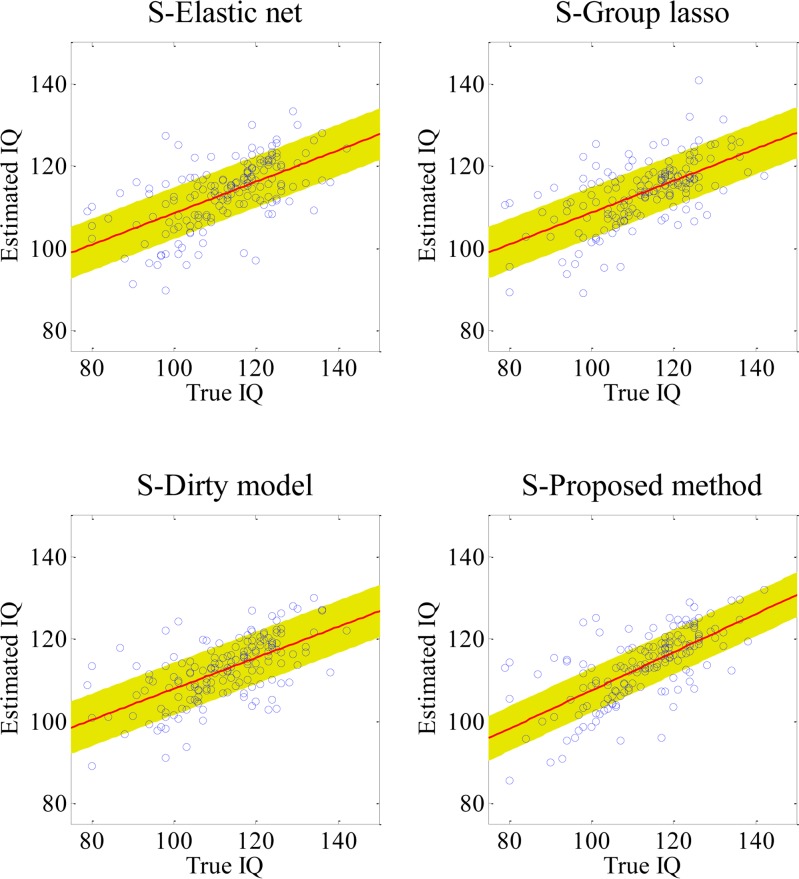
Scatter plots of the true IQ vs. the estimated IQ by single-kernel SVR. Scatter plots of the true IQ vs. the estimated IQ by the four competing methods with a single-kernel SVR, along with the standard deviation of the distance for each point to the fitted line.

In **Table 4**, the proposed method achieved the best CC of 0.684, while the other competing methods achieved 0.613 (S-Group lasso), 0.624 (S-Dirty model), and 0.598 (S-Elastic net). Here, we added a prefix ‘S’ in the front of the name of each method. The scatter plots of the true IQ scores and the estimated IQ scores are presented in **Fig. 6**. The proposed method achieved a RMSE of 9.166, outperforming the other competing methods: 9.763 (S-Dirty model), 9.905 (S-Group lasso) and 10.054 (S-Elastic net). Here, it is clear that the performance of other competing methods as well as the proposed method degraded with the use of a single-kernel SVR, compared to those with the use of a multi-kernel SVR. Among other competing methods, the performance of the group lasso degraded the least, while the performance of the elastic net degraded the most.

**Table 4:** Performance (mean ± standard deviation) comparison among all competing methods.

	S-Group lasso	S-Elastic net	S-Dirty model	S-Proposed method
CC	0.613 **±** 0.002	0.598 **±** 0.007	0.624 **±** 0.033	**0.684 ± 0.008**
RMSE	9.905 **±** 0.017	10.054 **±** 0.077	9.763 **±** 0.314	**9.166 ± 0.082**

The prefix ’S’ denotes the use of a single-kernel SVR. (CC: Correlation Coefficient; RMSE: Root Mean Square Error)

## Discussion

Because of the inapplicability of the current questionnaire-based IQ tests to the infants or young children, in this paper, we proposed a novel framework to estimate children’s IQ scores using structural MR images. To the best of our knowledge, this is a pioneering work for estimating a subject’s IQ score from neuroimaging data.

For neuroimaging data analysis, the high dimensionality of features overwhelms in general the number of samples available. Hence, dimension reduction or feature selection has been of great interest and of importance. In this paper, we use two types of features, i.e., GM and WM, and proposed a feature selection method for IQ score estimation. Since each GM feature and its corresponding WM feature are extracted from the same ROI, it is reasonable to assume that they are highly correlated, and also reasonable to utilize multi-task learning to incorporate the complementary information among different types of features [15,36]. Accordingly, we designed a new feature selection method based on a dirty model [24] with a newly devised regularization term, which can preserve advantages of the conventional dirty model but efficiently tackle the main disadvantage of the method, i.e., random selection of features from a group of highly correlated features.

To validate the proposed method, we performed two sets of experiments with the MRI data obtained from 164 typically developing children. In the first experiment, which focused on validating the efficacy of the proposed feature selection method by comparing with the state-of-the-art feature selection methods, our proposed method achieved the best performance with CC of 0.718 and RMSE of 8.695, outperforming all the comparison methods. We believe that this favorable performance was resulted from the well-designed regularization terms, allowing both group-wise and element-wise feature selection, as well as joint selection of a group of features that are highly correlated in a pairwise manner. Most of the regions selected by our method have been reported in previous studies and are highly associated with cognitive ability and memory. The selected regions include the right opercular part of the inferior frontal gyrus, the left hippocampus, the bilateral thalamus, and the bilateral transverse temporal gyri (Heschl's gyri). It has been found that the hippocampus, an important component in limbic system, play an important role in memory and spatial navigation [37,38], and thalamus is thought of as a switchboard of information that processes and relays the sensory information [39]. The inferior frontal gyrus is also found related to semantic task processing [40]. The Heschl’s gyri is found related to auditory processing and semantic task [41], and its abnormalities has been shown as one of the main reasons for the impairment of human cognitive abilities [42,43]. Since memory and cognitive abilities are the two important components that are commonly assessed in IQ tests [44], changes of GM/WM tissues in these ROIs may affect the quantification of human intelligence. In a supplementary experiment, we treat age as an independent type of features and further combine it with GM and WM features by using multi-kernel SVR, for the purpose of investigating whether it will affect the performance of our estimators. However, we did not observe any significant improvements compared to their counterparts only using WM and GM features.

In the second experimental paradigm, we proved the validity of assigning different weights to different feature types by comparing the estimators trained with a single-kernel SVR and a multi-kernel SVR. Again, the proposed method achieved better performances with CC of 0.684 and RMSE of 9.166 than the competing methods. However, the overall performances were degraded for all the methods compared to the case of using a multi-kernel SVR.

Because of the unavoidable variability among datasets scanned at different sites with different protocols and scanning parameters, we also designed a site classifier, which achieved an average classification accuracy of 98.5%, to identify the potential scanning site of a test image, before constructing multiple site-specific IQ score estimators. In our experiment with one general estimator built by the whole datasets, i.e., no consideration of scanning sites, the performances were 0.511 for CC and 10.873 for RMSE, which were much inferior to any of the methods via our site-specific estimator after identifying the scanning site. Here, it should be emphasized that our framework is not limited to estimate the test images scanned at one of the predefined sites. That is, in real application, the site classifier can play a role of identifying a scanning site, which has similar scanning parameters or protocols to the real scanning site of the test image.

## Conclusion

In this paper, we proposed a novel framework for the estimation of a subject’s IQ score based on the neuroimaging features. Methodologically, since the number of features in neuroimaging data usually overwhelms the number of available samples, feature selection has been always an important role in the field. To this end, considering the strong relationship between GM and WM features in MR images, we devised a feature selection method based on a dirty model [24] that efficiently considered the coupling of different feature types, but still alleviated the strong parameter overlap across features. Specifically, we penalized an objective function with a squared Frobenius norm of the element-wise sparsity matrix. Using the MR Images acquired at different scanning sites with their own scanning parameters and protocols, we designed a two-step procedure, by which we first identified the scanning site of a test image and then estimated the test subject’s IQ by using the respective estimator. Also, we performed comparison between multi-kernel SVR and single-kernel SVR by two sets of experiments. From a practical point of view, although the current framework is not limited to apply for the MR images obtained from only a predefined site, it would be our forthcoming research issue to develop a more generalized method for efficiently handling the inter-site variability and thus constructing a single generalized estimator model for all subjects by skipping the scanning site identification step. Furthermore, thanks to the availability of various imaging modalities, it would be beneficiary to integrate their complementary information for more precise IQ score estimation. It should be emphasized again that our work paves a new way for a research on *predicting* an infant’s future IQ score by using neuroimaging data, which can be a potential indicator for parents to prepare their child’s education if needed.
